# Long-Term Survival after Gamma Knife Radiosurgery in a Case of Recurrent Glioblastoma Multiforme: A Case Report and Review of the Literature

**DOI:** 10.1155/2012/545492

**Published:** 2012-04-04

**Authors:** Sudheer R. Thumma, Ameer L. Elaimy, Nathan Daines, Alexander R. Mackay, Wayne T. Lamoreaux, Robert K. Fairbanks, John J. Demakas, Barton S. Cooke, Christopher M. Lee

**Affiliations:** ^1^Gamma Knife of Spokane, Deaconess Health and Education Building, 910 W 5th Avenue, Suite 102, Spokane, WA 99204, USA; ^2^Cancer Care Northwest, 910 W 5th Avenue, Suite 102, Spokane, WA 99204, USA; ^3^MacKay & Meyer MDs, 711 S Cowley Street, Suite 210, Spokane, WA 99202, USA; ^4^Spokane Brain & Spine, 801 W 5th Avenue, Suite 210, Spokane, WA 99204, USA

## Abstract

The management of recurrent glioblastoma is highly challenging, and treatment outcomes remain uniformly poor. Glioblastoma is a highly infiltrative tumor, and complete surgical resection of all microscopic extensions cannot be achieved at the time of initial diagnosis, and hence local recurrence is observed in most patients. Gamma Knife radiosurgery has been used to treat these tumor recurrences for select cases and has been successful in prolonging the median survival by 8–12 months on average for select cases. We present the unique case of a 63-year-old male with multiple sequential recurrences of glioblastoma after initial standard treatment with surgery followed by concomitant external beam radiation therapy and chemotherapy (temozolomide). The patient was followed clinically as well as with surveillance MRI scans at every 2-3-month intervals. The patient underwent Gamma Knife radiosurgery three times for 3 separate tumor recurrences, and the patient survived for seven years following the initial diagnosis with this aggressive treatment. The median survival in patients with recurrent glioblastoma is usually 8–12 months after recurrence, and this unique case illustrates that aggressive local therapy can lead to long-term survivors in select situations. We advocate that each patient treatment at the time of recurrence should be tailored to each clinical situation and desire for quality of life and improved longevity.

## 1. Introduction

Glioblastoma multiforme (GBM) accounts for more than 50% of all gliomas and is the most aggressive type of primary brain tumor arising from glial cells. These tumors account for approximately 50% of all parenchymal brain tumor cases and 20% of all intracranial tumors. Approximately 10,000–25,000 cases are diagnosed annually in the United States [1, 2]. The peak incidence is around 45–55 years of age [3].

Despite advances in cancer therapy, treatment of glioblastoma remains a challenging issue and requires tailoring of treatment to each patient's clinical situation. Rarely are the available treatments curative, even with aggressive multimodality treatment regimens (surgery, radiation and chemotherapy) [4]. Studies have shown that the extent or completeness of resection has been correlated with survival [5, 6]. Upon initial diagnosis, the standard treatment consists of maximal surgical resection, fractionated external beam radiation (standard dose ranges between 5940 and 6000 cGy in 180–200 cGy per fraction), and concomitant and adjuvant chemotherapy with temozolamide. The progression-free survival with multimodality treatment is between 8 and 12 months. Despite multimodality treatment, the reported median survival is about 14–18 months [7].

Treatment options for recurrent GBM include chemotherapy, reirradiation, surgery, radiosurgery, antiangiogenic therapy, gene therapy, or best supportive care with corticosteroids [8, 9]. Treatment should be tailored to the individual situation, and the goal is to avoid the sequelae of iatrogenic neurotoxicity while maintaining a reasonable quality of life. Surgical resection is not an option for most patients in the recurrent setting, given the infiltrative nature of gliomas and proximity of the tumor to critical neurological structures. Reirradiation with conventional external-beam radiotherapy produces only a modest benefit in most patients, and in many situations, the risks outweigh the potential benefits [10]. Options are limited by the previous high doses of radiotherapy applied after the primary diagnosis, the tumor volume, and location at the time of recurrence. The response to systemic chemotherapy is also modest although new agents are being used such as bevacizumab (FDA approved for disease recurrence) [11]. Other options for treatment in the recurrent setting include combination or single agent chemotherapy, brachytherapy, and/or stereotactic radiation [12–16].

Stereotactic radiotherapy can be applied in a single fraction as stereotactic radiosurgery (SRS), or as fractionated stereotactic radiotherapy (FSRT) in which multiple fractions are given over a period of 2–4 weeks [16–20]. In this paper, we discuss the unique treatment results for a patient who was successfully treated with GK radiosurgery on three sequential occasions after recurrence and who survived for 7 years following the initial diagnosis.

## 2. Case Report

We present the unique case of a 63-year-old gentleman who initially presented with a five-week history of headaches behind the right eye, with associated “dizziness.” Two months prior to this, he had a syncopal episode. A CT scan at that time without contrast was reported as normal. On presentation, his wife noted that the patient needed help while getting dressed and was dragging his left leg, and he experienced weakness of the left hand. There were no complaints of nausea, vomiting, numbness, weakness, or any visual changes. His past medical history was significant for hypothyroidism and hyperlipidemia.

Physical examination of the patient revealed that he had a flat affect, his verbal responses were slow, and he was alert, oriented, and exhibited a flattened nasolabial fold on the left. Motor examination revealed equal strength and normal reflexes bilaterally in the upper arms, but slightly hypoactive patellar reflexes and ankles with downgoing plantars on the left. Cranial nerve examination was normal. A CT scan with and without contrast revealed a large, irregularly enhancing mass in the right temporal lobe that was associated with marked edema and mass effect, with mild uncal herniation and left shift suggestive of a brain tumor, suspicious of GBM.

Following the consultation with a neurosurgeon and discussing the possibility of a brain tumor and treatment options, the patient underwent a right temporal lobe lobectomy. The final pathology was consistent with GBM. The patient recovered well from surgery, and his headaches and dizziness were resolved with dexamethasone. Adjuvant treatment options were explained to him and included postoperative radiation with concurrent chemotherapy (temozolomide). The patient underwent radiation to a total dose of 6100 cGy with no complications. He took temozolomide only for a couple of months in the adjuvant setting, and this was discontinued due to side effects.

**Figure 1:**
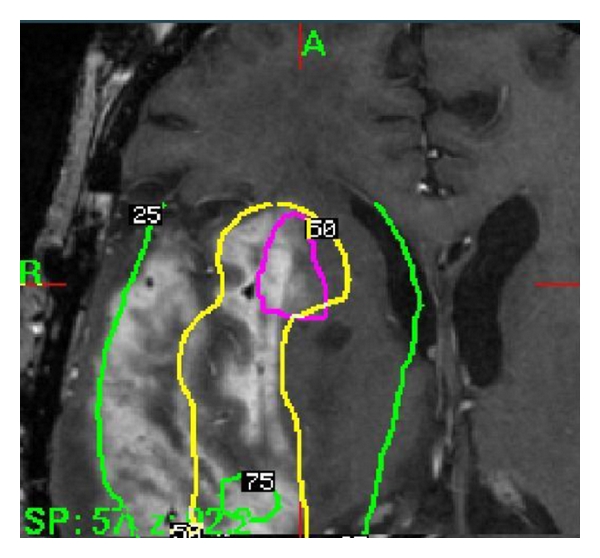
Gamma Knife treatment planning illustration of dose on an axial T1 postgadolinium MRI scan for the first Gamma Knife treatment.

One month after treatment, a followup scan revealed some residual enhancement in the operative bed consistent with residual/recurrent tumor versus radiation change. An MRI performed 12 weeks after treatment showed an increase in enhancement of the resection bed, and this was consistent with residual tumor. As the patient already had a full course of external beam radiation, the option of being treated with GK radiosurgery was presented, and the risks and benefits were discussed with the patient. The patient consented, and GK radiosurgery was successfully completed without complications. The marginal prescription dose was 16 Gy to the 45% isodose line for a 3.5 cm diameter region (see Figure 1).

Following his GK therapy, he was followed clinically as well as with surveillance MRI scans at 2-3-month intervals. Also, he was placed on monthly adjuvant Temozolomide, which was prescribed for 5 days of every 28-day cycles according to standard protocols. An MRI scan 4 months after his GK treatment again revealed an irregularly enhancing mass in the right temporal lobe which had enlarged significantly compared to the previous MRI two months prior. This was felt to represent recurrence and not radiation necrosis and was confirmed by review of this in light of past imaging studies with neuroradiology. The patient also described brief neurological symptoms at this time, with difficulty buttoning his shirt. However, this quickly resolved with dexamethasone therapy. Treatment options were again discussed and included repeat surgery (which would have been technically difficult and would have been associated with significant risks) versus undergoing repeat GK radiosurgery. The patient consented to repeat GK and successfully underwent the procedure a second time without complications. The second GK dose prescribed to the 50% isodose line was 14 Gy to a 4.2 cm diameter region.

**Figure 2:**
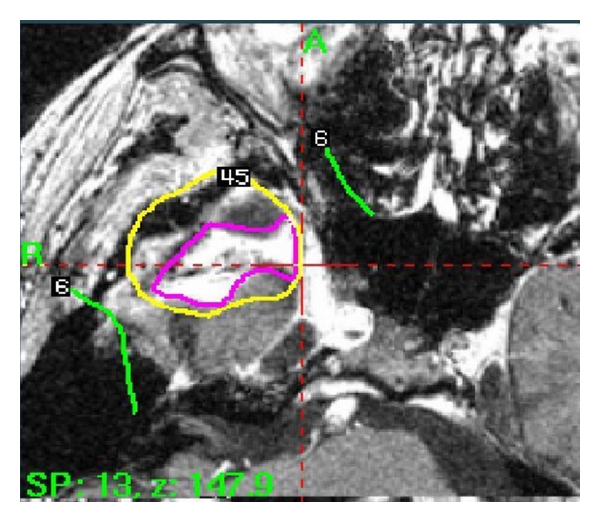
Gamma Knife treatment planning illustration of dose on an axial T1 postgadolinium MRI scan for the third Gamma Knife treatment.

Again, the patient was followed with serial MRI scans at two- to three-month intervals as well as clinically and again was found to have radiographic documentation of disease recurrence 14 months following his last treatment. He again consented to a third treatment with GK. For his third GK treatment, the dose prescribed to the 50% isodose line was 12 Gy to a 4.9 cm diameter region (see Figure 2). Following his third and final GK treatment, the patient survived a total of 69 months. During the final years of his life, the patient developed dizziness, confusion, visual problems, seizures, and weakness. He also developed new onset diabetes for which he was put on insulin. The patient gradually became wheel chair bound and later developed lower extremity DVT. He was not anticoagulated because of increased risk of cerebral bleeding due to the infiltrating and enlarging nature of the tumor. Recommendation was made for hospice, but the patient refused and later succumbed to his disease at home.

## 3. Discussion

Although the prognosis of patients with glioblastoma is uniformly poor, treating the patients in an attempt to improve the quality of life and longevity is of utmost importance. Variables predicting longer survival in several studies include extent of resection (gross total versus subtotal), younger age (<50 years), higher Karnofsky performance scores (KPS), normal mental status, and smaller tumor volume [21, 22]. MGMT methylation is also associated with longer survival, and a testing option for this is commercially available [23]. Amplification and activating mutations of the epidermal growth factor receptor (EGFR) oncogene are also common in glioblastomas [24, 25]. However, much remains unknown about why some patients survive longer with glioblastoma [26–29].

Many chemotherapeutic agents, either as single agents or in combination chemotherapy regimens, have been used to treat recurrent malignant gliomas [30, 31]. Temozolomide, PCV, bevacizumab are some of the treatment options [32–35]. Bevacizumab has been approved by FDA for the treatment of recurrent GBMs [11]. Temsirolimus (CCI-779), a small-molecule inhibitor of the mammalian target of rapamycin (mTOR), has shown activity in recurrent gliomas [36]. Erlotinib and other EGFR inhibitors have been studied in combination with radiation therapy [37, 38].

Given that recurrence has been postulated to be secondary to persistent stem cells, vaccine therapy has been attempted in relapsed cases [39]. Small trials have reported that a tumor B-cell hybridoma vaccine against tumor stem cells elicited a specific tumor immune reaction thus enhancing immune response to the disease [40]. Larger trials are in progress to further assess this approach to treating glioblastoma.

Brachytherapy for recurrent disease using high-activity I-125 interstitial implants has been shown to improve median survival in highly selected patients to around 8 months [13, 35]. Glia-Site brachytherapy, using an inflatable balloon catheter implanted into the surgical resection cavity for delivery of homogeneous low-dose rate radiation, has also shown promising results in patients with recurrent gliomas [41]. Radiation in the form of brachytherapy is highly invasive; thus, this technique is often associated with high morbidity and therefore limited to small subgroup of patients.

SRS has the ability to precisely deliver high doses of radiation to a defined tumor volume in a single fraction with less treatment-associated morbidity compared with surgery. FSRT involves delivering radiation to a defined tumor volume, exploiting the radiobiologic advantage of fractionation, and thus minimizing the risk of serious side effects [42]. Stereotactic external beam radiotherapy can be applied in single fractions as SRS or as FSRT in which multiple fractions are given over a period of 2–4 weeks. With this approach, the dose within the tumor is much higher than the dose in the surrounding normal brain tissue, as a result of sharp dose gradient achieved by multiple converging beams of radiation. The three most common types of radiation therapy devices used are Cobalt-60-based machines, linear accelerators, and cyclotrons which use gamma rays, X-rays, and protons, respectively.

The RTOG conducted a multicentered randomized phase III trial analyzing the addition of SRS to standard external beam radiation therapy (EBRT) for the treatment of glioblastoma. In this trial, SRS was used as part of the initial management rather than as a salvage therapy. 203 patients with supratentorial glioblastoma were randomly assigned to receive either postoperative SRS followed by EBRT and carmustine, or EBRT and carmustine without SRS. They observed no statistically significant differences between the two groups of patients with regard to the primary endpoint of survival around 13.5 months [43]. However, a number of institutional reviews have been reported that show encouraging results including survival prolongation in the recurrence setting for specific patients. The timing of SRS and lack of careful patient selection may explain the apparent lack of benefit in this setting. Patients are selected based on age, performance status, extracranial volume of disease, intracranial tumor volume, and by histologic type and number of brain metastases. We utilize a standard treatment algorithm at our institution based on tumor size, proximity of nearby critical structures, and prior radiation treatment received. This algorithm is adapted to published clinical research on radiation doses, volumes, and accepted treatment techniques.

Combs et al. reported long-term results in 172 patients treated in a single institution highlighting the efficacy of fractionated stereotactic reirradiation in recurrent gliomas [19]. Between 1990 and 2004, 172 patients with recurrent gliomas were treated with FSRT as reirradiation. Approximately 59 patients were diagnosed with GBM, and the median time between primary radiotherapy and reirradiation was 10 months for GBM. FSRT was performed with a median dose of 36 Gy in a median fractionation of 10 Gy/wk. Median overall survival after primary diagnosis was 21 months for patients with glioblastoma. Time to progression and histology were significant factors influencing survival after reirradiation. Progression-free survival after FSRT was 5 months. Villavicencio et al. reported their experience with twenty patients who underwent Cyber Knife treatment at the time of the initial diagnosis and/or during the first 3 months of their initial clinical management [44]. Twenty six patients were treated at the time of tumor recurrence or progression. Cyber Knife was performed in addition to the traditional therapy. The median survival from diagnosis for the patients treated with Cyber Knife as an initial clinical therapy was 11.5 months (range, 2–33) compared to 21 months (range, 8–96) for the patients treated at the time of tumor recurrence/progression. This difference was reported to be statistically significant (Kaplan-Meier analysis, *P* = 0.0004). The median survival from the Cyber Knife treatment was 9.5 months (range, 0.25–31 months) and 7 months (range, 1–34 months) for patients in the newly diagnosed and recurrent GBM groups (Kaplan-Meier analysis, *P* = 0.79), respectively. Patients with more extensive surgical interventions survived longer (*P* = 0.008), especially those who underwent total tumor resection versus biopsy (*P* = 0.004). This revealed that there was no apparent survival advantage in using CyberKnife in the initial management of glioblastoma patients, and it should be reserved for patients whose tumors recur or progress after conventional therapy.

The above trials show the effectiveness of SRS as an option for the treatment of recurrent glioblastoma. Effective implementation of stereotactic radiosurgery requires a team of well-trained neurosurgeons, radiation oncologists, medical oncologists, and medical physicists who work together as a part of multidisciplinary team. GK requires localization and immobilization of the target with the attachment of head frame to the skull. With local anesthesia, four screws pierce the scalp and secure the frame to the outer table of the skull. MRI is then performed to localize the target(s) in three dimensions. With the help of high-speed computers, physicians and the physicists develop a treatment plan that focuses the radiation as precisely as possible on the tumor target. After the treatment is complete, the head frame is removed, and the patient is usually discharged within an hour. Some patients may require pain medications after the frame removal, which occasionally delays discharge. Many patients are able to resume routine activities within a week after the procedure. The followup of patients at our center consists of an MRI after a month and thereafter every 2-3 months to assess for local control and tumor recurrence.

## 4. Conclusion

This is the first reported case to our knowledge of a patient with recurrent glioblastoma who survived seven years following treatment with GK radiosurgery. The patient underwent salvage treatment two additional times during the 7 year period and tolerated the therapy well. This case highlights the unique biology of individual tumors and that focused radiation in the recurrent setting can significantly impact overall survival for specific patients. We look forward to continued research in this area as well as into the molecular characteristics of individual tumors. Continued individualization of treatment for each unique patient's situation will allow for improvements in survival as well as quality of life.
